# Risk assessment, surveillance, and nonpharmaceutical prevention of acute radiation dermatitis: results of a multicentric survey among the German-speaking radiation oncology community

**DOI:** 10.1007/s00066-023-02074-w

**Published:** 2023-04-26

**Authors:** Katharina Layer, Julian P. Layer, Andrea R. Glasmacher, Gustavo R. Sarria, Alexander M. C. Böhner, Yonah L. Layer, Cas S. Dejonckheere, Stephan Garbe, Petra Feyer, Brigitta G. Baumert, Anke Schendera, René Baumann, David Krug, Mümtaz A. Köksal, David Koch, Davide Scafa, Christina Leitzen, Michael Hölzel, Frank A. Giordano, Leonard Christopher Schmeel, the German-Speaking Radiation Dermatitis Survey Group

**Affiliations:** 1 Department of Radiation Oncology, University Hospital Bonn, University of Bonn, Venusberg-Campus 1, 53127 Bonn, Germany; 2 Institute of Experimental Oncology, University Hospital Bonn, University of Bonn, Bonn, Germany; 3 Department of Radiation Oncology, Vivantes Hospital Neukölln, Berlin, Germany; 4 Institute of Radiation Oncology, Cantonal Hospital Graubünden, Graubünden, Switzerland; 5 Department of Radiation Oncology, Community Hospital Mittelrhein, Koblenz, Germany; 6 Department of Radiation Oncology, St. Marien Hospital Siegen, Siegen, Germany; 7 Department of Radiation Oncology, University Medical Center Schleswig-Holstein, Kiel, Germany; 8 Department of Radiation Oncology, University Hospital Mannheim, University of Heidelberg, Heidelberg, Germany

**Keywords:** Radiation dermatitis management, Wound management, Prophylaxis, Risk factors, Radiation side effects

## Abstract

**Purpose:**

Radiation dermatitis (RD) represents one of the most frequent side effects in radiotherapy (RT). Despite technical progress, mild and moderate RD still affects major subsets of patients and identification and management of patients with a high risk of severe RD is essential. We sought to characterize surveillance and nonpharmaceutical preventive management of RD in German-speaking hospitals and private centers.

**Methods:**

We conducted a survey on RD among German-speaking radiation oncologists inquiring for their evaluation of risk factors, assessment methods, and nonpharmaceutical preventive management of RD.

**Results:**

A total of 244 health professionals from public and private institutions in Germany, Austria, and Switzerland participated in the survey. RT-dependent factors were deemed most relevant for RD onset followed by lifestyle factors, emphasizing the impact of treatment conceptualization and patient education. While a broad majority of 92.8% assess RD at least once during RT, 59.0% of participants report RD at least partially arbitrarily and 17.4% stated to classify RD severity solely arbitrarily. 83.7% of all participants were unaware of patient-reported outcomes (PROs). Consensus exists on some lifestyle recommendations like avoidance of sun exposure (98.7%), hot baths (95.1%), and mechanical irritation (91.8%) under RT, while deodorant use (63.4% not at all, 22.1% with restrictions) or application of skin lotion (15.1% disapproval) remain controversial and are not recommended by guidelines or evidence-based practices.

**Conclusion:**

Identification of patients at an increased risk of RD and subsequent implementation of adequate preventive measures remain relevant and challenging aspects of clinical routines. Consensus exists on several risk factors and nonpharmaceutical prevention recommendations, while RT-dependent risk factors, e.g., the fractionation scheme, or hygienic measures like deodorant use remain controversial. Surveillance is widely lacking methodology and objectivity. Intensifying outreach in the radiation oncology community is needed to improve practice patterns.

**Supplementary Information:**

The online version of this article (10.1007/s00066-023-02074-w) contains supplementary material, which is available to authorized users.

## Introduction

Disruption of the epidermal barrier function is a physiological reaction to ionizing radiation exposure and, as such, an undesirable but inevitable side effect of external-beam radiation therapy (RT). Hence, radiation dermatitis (RD) represents the most common side effect of RT [1], occurring in up to 95% of patients, depending on tumor entity [2–4]. While technical advances have been able to significantly reduce overall RD severity [4], identification of risk factors remains crucial for an aging patient collective exposed to increasingly intensified systemic and RT treatment regimens.

Symptoms of RD mainly depend on deterministic factors [5, 6]. Acute RD is mostly self-limiting within several weeks and characterized by erythema, edema, dry or moist desquamation, and pain [7]. Chronic tissue changes might follow acute RD or occur after a latency period [8] and may manifest as cutaneous fibrosis, telangiectasia, pigmentation, atrophy, or even skin necrosis [9]. RD may considerably affect quality of life (QOL) [4] and even lead to discontinuation of effective treatments [10].

Primary prophylaxis with comprehensive patient education, counseling, and standardized objectified assessments allowing for early diagnosis of RD can prevent or at least delay pharmaceutical interventions. To this end, uniform recommendations must be directed not only to radiation oncologists but also to associated staff and involved clinical stakeholders [11]. Various studies have pointed towards high levels of heterogeneity and bias in skin care practices among radiation oncologists, presumably due to anecdotal evidence [12–18]. However, previous reports on German-speaking Europe have focused mainly on pharmaceutical prevention and treatment of RD [19, 20]. It is to date unknown what practice patterns for RD assessment and nonpharmaceutical prevention are common in the German-speaking community of radiation oncologists.

Hence, we carried out a survey to characterize risk factors for RD, daily practice surveillance, and to comprehensively investigate current nonpharmaceutical practice patterns for prevention in the German-speaking radiation oncology community.

## Materials and methods

### Questionnaire

A questionnaire encompassing 36 items was developed (Supplementary Appendix). Thematic subdivisions were general personal data, RD diagnostics and surveillance, and prevention and treatment of RD. This report focuses on both the surveillance and prevention segments of the questionnaire. For each question, a comprehensive list of different predefined approaches was prepared. Multiple choices were allowed to gain insights into the entire range of therapies offered at each institution. Individually defined Likert scales were arbitrarily designed, ranging from a lowest to the highest ordinal category available for selection. Participants were generally asked for a ranking of said preselected items. Free-text answers were analyzed separately, if available. All personal information was anonymized prior to analysis. In case of incomplete surveys, any blank fields were excluded from the analysis. Participants were able to opt against annotation of their names and affiliations in order to partake in the German-Speaking Radiation Dermatitis Survey Group (GRDSG).

### Technical implementation

As an online survey interface and data storage server, the Findmind software (Findmind Online Umfragen, St. Gallen, Switzerland) and website were used. The survey was accessible through an invitation link sent by email on December 2, 2021, to all members of the German Society for Radiation Oncology (DEGRO) who had given prior consent to receive science-related content. A reminder email was sent on January 12, 2022. The survey was closed on January 21, 2022.

### Statistical analysis

Data analysis was performed with R [21] and GraphPad Prism 9 (GraphPad Software, USA). If not stated otherwise, statistical tests and analyses were performed as indicated in the respective figure legends. Figures were generated using GraphPad Prism 9 and Adobe Illustrator 2021 (Adobe Inc., USA).

## Results

**Fig. 1:**
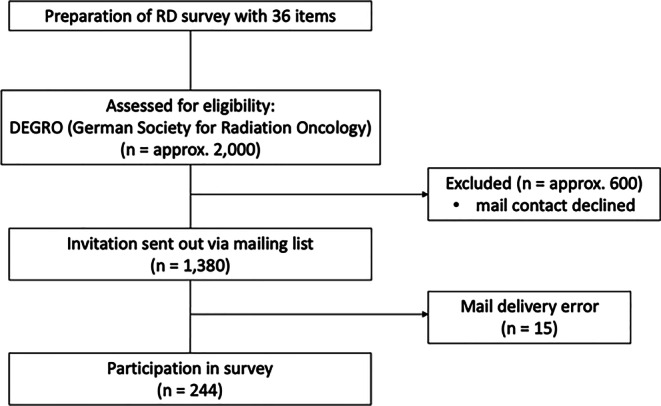
Survey outline

The survey was sent to 1,380 DEGRO members via email (Fig. 1). With a response rate of 17.9%, a total of 244 DEGRO members participated in the survey. Four participants were located in Austria, four in Switzerland, and 236 in Germany. The majority of responders were employed at academic institutions (academic hospital clinicians [AHC]). The second largest group was employed at private centers (private center clinicians [PCC]), followed by almost equally represented nonacademic hospital clinicians (NAC).

Most participants were chief or senior physicians, followed by board-certified radiation oncologists and residents. Medical physicists, technical assistants, nurses, or physician assistants accounted combined for 4.6%. The largest represented age cohort was between 51 and 60 years of age (Table 1).

**Table 1:** Participant characteristics

Characteristic	%
*Sex*	
Male	43.5
Female	57.5
*Age (years)*	
20–30	12.8
31–40	27.2
41–50	19.4
51–60	31.7
> 60	8.9
*Institution*	
Academic hospital	40.7
Nonacademic hospital	28.8
Private Center	29.4
Others	1.1
*Experience*	
Senior physicians	43.1
Board-certified physicians	27.6
Residents	24.1
Assistants/nurses	2.9
Physicists	0.6
Others	1.7

**Fig. 2:**
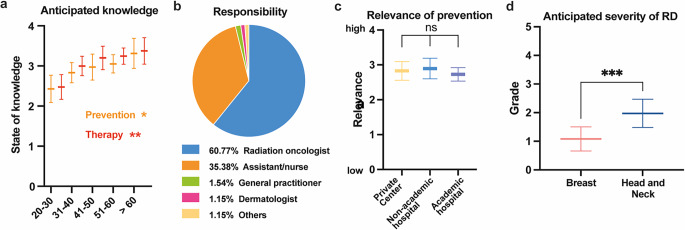
General opinion of participants on radiation dermatitis (*RD*) management. **a** Mean self-assessed knowledge on RD prevention (*orange*) and therapy (*red*) by age subgroups. Error bars indicate 95% confidence intervals (CI); *p < 0.05, **p < 0.01, analysis of variance (ANOVA). **b** Pie chart of staff considered responsible for RD management. **c** Mean relevance of prevention by institution subgroups. Error bars indicate 95% CI; *p* > 0.05. **d** Mean subjective severity of RD by CTCAE grade in breast (*red*) and head and neck (*blue*) cancer. Error bars indicate standard deviation; ***p < 0.001, Welch’s *t*-test

The participants felt equally well informed about both state-of-the-art prevention and treatment of RD (*p* > 0.05; mean 2.98 ± 0.78 and 3.13 ± 0.74, respectively). PCC considered themselves to be slightly better informed than NAC and AHC (*p* > 0.05). Self-assessed knowledge significantly increased with age (Fig. 2a). There was general consensus that prevention and management of RD are primarily responsibilities of radiation oncology staff (Fig. 2b), i.e., either physicians themselves (60.8%) or specialized nurses (35.4%). The relevance of RD prevention in clinical routine was considered high, with an average rating of 2.83 ± 0.91 on a four-item Likert scale (Fig. 2c). Here, NAC deemed this topic to be of higher relevance than did AHC or PCC. However, this difference was not significant (*p* = 0.72). The mean observed RD severity was reported as Common Terminology Criteria for Adverse Events (CTCAE) grade 1.08 for breast and 1.97 for head and neck cancer (Fig. 2d).

**Fig. 3:**
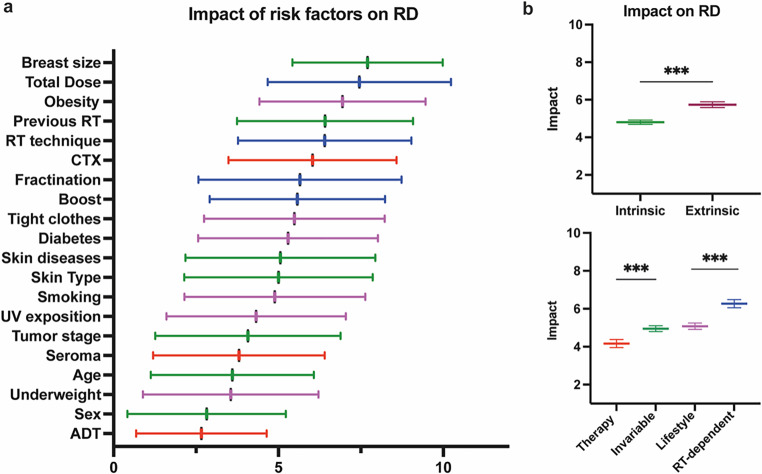
Impact of risk factors on radiation dermatitis. **a** Mean considered impact of listed risk factors color-coded by treatment-associated (*red*), invariable (*green*), lifestyle (*purple*), and RT-dependent (*blue*) risk factors. Error bars indicate 95% confidence intervals (CI). **b** *Upper panel:* mean considered impact of risk factor groups grouped by intrinsic (*green*) and extrinsic (*red*) factors. *Lower panel:* mean considered impact of risk factor groups grouped as in **a**. Error bars indicate 95% CI; ***p < 0.001, Welch’s *t*-test. *ADT* androgen deprivation therapy, *CTX* chemotherapy, *RD* radiation dermatitis, *RT* radiotherapy

Several risk factors were considered relevant for the development of RD. Intrinsic factors are individual constitutions including body mass index or preexisting skin diseases [22], whereas extrinsic factors are treatment-related conditions, i.e., adjuvant treatment [23, 24], use of bolus, or RT technique [25–29]. The highest mean impact was attributed to breast size, with 7.7 on a scale from 1 to 10 (Fig. 3a). Total dose of RT (7.45), obesity (6.94), previous local RT (6.41), RT technique (6.4), and chemotherapy (6.03) were also deemed highly correlative to RD severity by the respondents. Supplementary Fig. 1 shows the variance of the respective values depicting the degree of concordance between participants. With a variance of 9.46, the impact of the fractionation scheme was by far the most controversial risk factor. Interestingly, grouping of the associated risk factors revealed a significantly higher impact of extrinsic than of intrinsic factors (Fig. 3b). RT-dependent factors were followed by lifestyle factors, emphasizing the impact of treatment conceptualization and patient education.

**Fig. 4:**
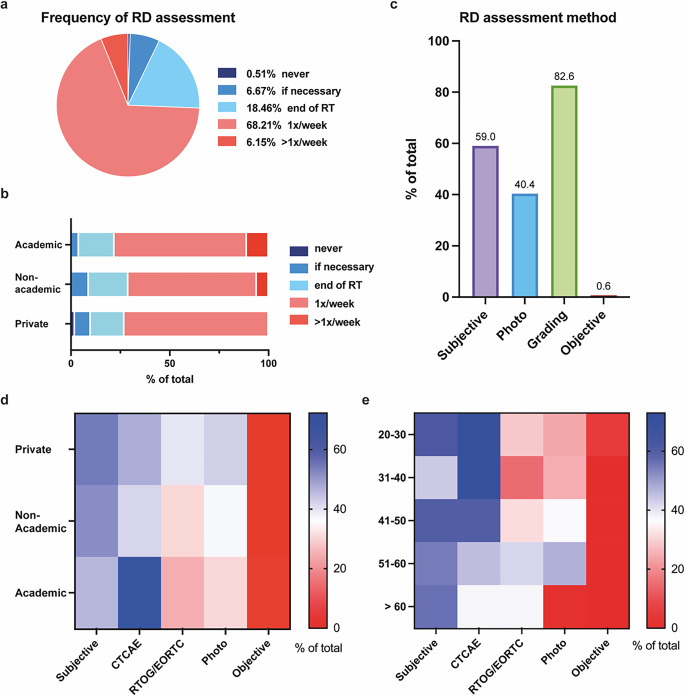
Radiation dermatitis (*RD*) assessment. **a** Pie chart of RD assessment frequency. **b** Frequency as in **a**, but grouped by institution. **c** RD assessment method of choice. More than one answer was possible. **d**, **e** Double-gradient heatmaps showing RD assessment method of choice across institutions (**d**) and age (**e**) from 0% (*red*) to 75% (*blue*) of total

Participants uniformly stated regular recording of RD during RT (Fig. 4a): 92.8% assess RD at least once during RT, regardless of symptom burden; 68.2% record RD once per week; and 6.2% even two to five times per week. RD is recorded more frequently by AHC than by PCC (Fig. 4b). 59% of participants report RD at least partially subjectively, while 17.4% of participants stated to classify RD severity solely subjectively (Fig. 4c). 82.6% use validated scales such as the NCI CTCAE or the Radiation Therapy Oncology Group (RTOG)/European Organization for Research and Treatment of Cancer (EORTC) criteria. Other response options such as skin toxicity assessment tool (STAT), skin index 16, radiation-induced skin reaction assessment scale, or brief pain inventory were not provided by the participants. 40.4% regularly perform photo documentation and less than 1% use objective measurements like reflection spectrophotometry or laser Doppler flowmetry. AHC and NAC proved more likely to make use of advanced and more precise documentation than PCC; however, this difference was not significant.

Whereas 83.7% of all participants were unaware of any availability of patient-reported outcomes (PROs), of those few participants who were aware of PROs, 74.1% performed PRO assessments weekly. 69.2% used individual/arbitrary scales for documentation, 23.1% used the PRO-CTCAE grading.

AHC and participants of younger age were more likely to use CTCAE gradings (Fig. 4d, e), whereas PCC and participants of older age rather employed the RTOG/EORTC grading system. PCC and NAC were more likely to conduct subjective/arbitrary and photograph-based documentations. 61.6% of participants always record anamnestic skin diseases prior to RT, while 30.2% assess preexisting skin diseases irregularly (Supplementary Fig. 2). Topical medication is regularly recorded by 51.6% and irregularly by 41.9%. 84.9% do not classify skin types by any scale.

**Fig. 5:**
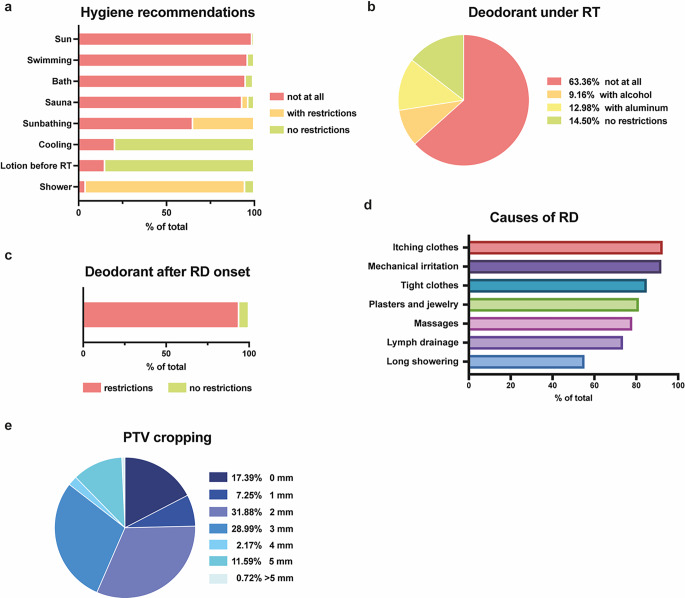
Hygiene recommendations and nontherapeutic prophylaxis of radiation dermatitis (*RD*). **a** Restrictions in hygiene procedures and activities. **b** Pie chart of deodorant usage under radiotherapy (*RT*). **c** Deodorant use after RD onset. **d** Pie chart of planning target volume (*PTV*) cropping in millimeters. **e** Habits and procedures considered as causes of RD

There was consensus regarding hygiene measures for RD prevention (Fig. 5a). Participants widely agree that sun exposure (98.7%), swimming (96.2%), or bathing (95.1%) should be avoided under RT. Equally, only 3.5% generally allow sauna visits and a similar proportion recommend ceasing them after RD onset. Sunbathing was restricted to the skin area not affected by RT by 34.9%, whereas the remaining 65.1% recommended to avoid it overall during RT. Skin cooling (79.3%) and cream or lotion application right before RT (84.9%) are mostly allowed. 90.7% recommend using mild, pH-neutral, and fragrance-free shower gel, while a minority of 4% disapprove of showering during RT completely. Additionally, a majority advise limiting the length of showers to the necessary. Only 14.5% allow use of deodorants unconditionally, while 13% recommend restricting usage to deodorant containing aluminum but no alcohol and another 9.2% recommend the opposite, i.e., preparations with alcohol but no aluminum (Fig. 5b). 94% stated that deodorant application should be withheld at the onset of RD (Fig. 5c).

For most participants, mechanical irritation of the skin should be avoided by all means (Fig. 5d). To a lesser extent, massages and lymph drainage are not recommended.

With 82.6% of participants, most institutions appear to reduce skin toxicity by cropping the cutis from the planning target volume (PTV), although various participants report sparing from 1 to more than 5 mm of PTV of the skin (Fig. 5e). There was no significant difference between PCC (mean PTV cropping 2.37 mm), NAC (1.92 mm), and AHC (2.47 mm).

## Discussion

Modern RT techniques are often associated with reduced peak skin doses and reduced RD severity. However, depending on tumor entity and treatment regimen, mild and moderate RD still affects major subsets of patients in clinical routine. A focus on and management of patients with a high risk of severe RD is even more essential and requires profound a priori risk assessment. This survey identified several risk factors deemed highly relevant by the community. As reported by our findings, broad consensus has been reached on many of these RD risk factors and aspects of nonpharmaceutical prevention of RD. However, certain aspects remain controversial for at least a minority of the German-speaking radiation oncology community. The surveillance and assessment of RD is heterogeneous and still driven by personal preference and experience rather than by empiric data.

We found that AHC are more likely to assess skin status preventively, whereas PCC tend to react at the onset of RD. A possible explanation for this might be that there is still no gold standard for RD assessment due to lack of reliability, validity, and consistency [30, 31]. Accurate monitoring and classification of RD is thus essential for adequate prophylaxis and treatment decisions. The most common tools for classifying skin toxicity are the CTCAE or the RTOG/EORTC grading. While these systems differ only slightly, an advantage of the RTOG/EORTC classification might be the assessment of both acute and chronic RD. It is, however, less commonly used compared to CTCAE [32]. Most participants rely on subjective reporting of RD. Standardized documentation of RD is increasingly applied, but there are limited data on its validation and reliability [6, 33, 34]. Physician-assessed symptoms often underestimate the severity of RD compared to patients themselves, leading to discrepancies between clinician-reported outcomes and PROs [34, 35]. Therefore, the awareness of PROs for assessment in clinical trials and also in clinical routine is steadily increasing [36, 37]. Of note, in this survey, an unexpectedly high proportion (12.1%) assess PROs weekly, although the vast majority of participants is still unfamiliar with the concept of PROs. For these reasons, future RD assessment tools should be critically scrutinized and include PROs. Besides outcome reporting, patients should also be actively included in RD prevention strategies.

Studies investigating prognostic factors for RD in breast cancer patients suggest age, high body mass index (BMI), and large breasts to play an important role in the development of acute skin toxicity during RT [34, 38]. This is mostly in line with participants’ opinions, although the impact of age was rather underestimated. Besides intrinsic factors, extrinsic factors such as dose fractionation [6, 34], treatment technique, or concomitant therapies [39, 40] affect RD incidence and severity. Our variance analysis showed that there was considerable discrepancy regarding the role of fractionation schemes, which reveals a substantial uncertainty of the community in handling one of their most influential impact factors on RD risk. As the factors deemed most relevant in this survey are modifiable by the patient or radiation oncologist, our results underscore the significance of identifying patients at risk prior to RD and raising awareness among both patients and clinicians.

Surprisingly, despite the existing guidelines and recommendations [41], 4% still recommend avoiding showering during RT, which may derive from former times when Co^60^ devices were widely available and, thus, RD was more frequent and severe [42]. As confirmed for various cancer entities and treatment regimes, washing irradiated skin with soap does not cause adverse skin reactions [13, 42–49]. Discouraging patients from daily washing can be unnecessarily stressful [50], impair QOL [51], and should be avoided. Likewise, prohibiting deodorants during RT is of unproven benefit and does not decrease RT toxicity [33, 52–54], but does have a negative impact on QOL [55, 56]. Nonetheless, 63.4% of our respondents disapprove of deodorant use during RT. We found a slight age dependency regarding approval of deodorant usage under RT. This might be based on the assumption of a possible bolus effect of deodorant [57, 58] interactions between metal particles and radiation [59] or a skin-irritating effect of alcohol. One out of six radiation oncologists in this survey prohibits application of lotion before RT. However, patients can continue their usual skin care regimen without experiencing increased toxicity [48, 52, 60].

Most participants perform a skin sparing PTV cropping to help reach adequate conformality while reducing skin toxicity. In the absence of skin infiltration, this is certainly useful for optimization of dose coverage statistics, but rather questionable for reducing the actual skin exposure. Due to a wide range of PTV cropping margins used, no trend has emerged as to how much PTV should actually be subtracted from the skin. This is not surprising as there are no studies investigating and comparing the efficacy of PTV skin-sparing techniques in RD prevention yet.

There are some limitations to this study. Certain health care professionals directly involved in RT, in particular physicists, nurses, and radiation therapists, are rather underrepresented. Contrarily, more experienced physicians in leading positions are overrepresented. Additionally, the relatively higher academic background may constitute a participation bias, but the presumably higher experience of the participants also strengthens the validity of the data obtained. This survey was designed to provide a comprehensive overview of current RD risk management and RD prevention approaches independent of particular tumor entities or body regions. Therefore, the survey cannot account for all entity-specific issues and treatment features. Even though this survey cannot cover the entire scope of approaches, it represents the most comprehensive within the German-speaking radiation oncology community to date.

## Conclusion

Risk stratification, early recognition, and prevention of RD remain fundamental in radiation oncology, which is subject to constant change as technical procedures and concomitant therapies evolve. Despite all progress made in recent years, substantial differences persist amongst practitioners regarding identification of RD risk factors, patient counseling, surveillance, and nonpharmaceutical management. RD surveillance is widely lacking methodology and objectivity. While a general consensus has been partially reached with regard to patient counseling and nonpharmaceutical prevention of RD, specific aspects reveal institutional differences and knowledge gaps. Identifying these gaps eases planning of interventional strategies and is compulsory to tailor patient-centered, interdisciplinary prevention in homogenized health care patterns.

### Supplementary Information

Supplementary Appendix: Survey

Supplementary Table 1: Members of the GRDSG

Supplementary Fig. 1: Variance in impact of risk factors on radiation dermatitis color-coded by treatment-associated (red), invariable (green), lifestyle (purple) and RT-dependent (blue) risk factors. Abbreviations: ADT = androgen deprivation therapy; CTX = chemotherapy; RD = radiation dermatitis; RT = radiotherapy.

Supplementary Fig. 2: Radiation dermatitis-relevant anamnesis.
