# Supplementary material for: Risk assessment, surveillance, and nonpharmaceutical prevention of acute radiation dermatitis: results of a multicentric survey among the German-speaking radiation oncology community
Source: Strahlenther Onkol. 2023 Apr 26;199(10):891–900. doi: 10.1007/s00066-023-02074-w (PMC10542714; doi:10.1007/s00066-023-02074-w)
Supplement: Supplementary file 1 — Supplementary Appendix: Survey [file 66_2023_2074_MOESM1_ESM.pdf]

Suppl. 1

1 - Freiwillig: Geben Sie Ihren Namen und Ihre Institution(en) an.

Name, Vorname(n)

Institution(en), bitte mit Anschrift

2 - Auf geht's - viel Spaß beim Beantworten der Fragen!
*Anm.: Bei der Beschreibung der Patient*innen haben wir wegen des besseren Leseflusses nur ein Geschlecht ausgeschrieben. Selbstverständlich sind damit stets Patienten jeglichen Geschlechts gemeint.*

3 - An welchem Ort sind Sie tätig?
Stadt, Land
z.B. Bonn, Deutschland

---

4 - In was für einer Einrichtung sind Sie tätig?

◯ Praxis/Gemeinschaftspraxis/Praxisverbund/MVZ
◯ Krankenhaus/Klinik (ggf. mit angebundenem MVZ)
◯ Universitätsklinik (ggf. mit angebundenem MVZ)
Andere Art von Einrichtung:

---

5 - Wie alt sind Sie?
◯ 20-30
◯ 31-40
◯ 41-50
◯ 51-60
◯ 60 +

6 - Welche Funktion üben Sie in Ihrer Einrichtung aus?
◯ Chefarzt/Leitender Arzt/Oberarzt /-ärztin
◯ Facharzt /-ärztin
◯ Assistenzarzt /-ärztin
◯ Medizinphysiker /-in
◯ MTRA, Krankenpfleger, Arzthelfer /-in
Andere:

---

7 - Wie gut schätzen Sie Ihren Informationsstand zur Prophylaxe und Therapie der Radiodermatitis ein?
Bitte geben Sie uns Ihre authentische Einschätzung (vier Sterne für sehr gute Kenntnisse).

**zur Prophylaxe**	|-----------------------O-----------------------|
**zur Therapie**	|-----------------------O-----------------------|

8 - Führen Sie aktuell Studien zur Prophylaxe/Behandlung der Radiodermatitis durch?

◯ ja
◯ nein
◯ in Vorbereitung

9 - Wer sollte Ihrer Meinung nach für die Prophylaxe und Therapie der Radiodermatitis primär zuständig sein?

☐ Strahlentherapeut /-in
☐ (geschulte) MTRA bzw. Pflegekraft (Wundmanager /-in)
☐ Hausarzt /-ärztin
☐ Dermatologe /-in
Andere:

---

10 - Welchen Stellenwert besitzt die Prophylaxe der Radiodermatitis in Ihrer klinischen Routine?

**bis maximal vier Sterne für höchste Relevanz**	|-----------------------O-----------------------|

11 - Was sind Ihrer Meinung nach relevante Einflussfaktoren für eine Radiodermatitis:
Je wichtiger der Einflussfaktor, umso mehr schieben Sie den Slider nach rechts.

**(anti-)hormonelle Therapie**	|-----------------------O-----------------------|
**Adipositas**	|-----------------------O-----------------------|
**Alter**	|-----------------------O-----------------------|
**Bestrahlungstechnik (3DCRT vs. IMRT/VMAT)**	|-----------------------O-----------------------|
**Brustgröße**	|-----------------------O-----------------------|
**Boost bei Brustbestrahlungen**	|-----------------------O-----------------------|
**Chemotherapie**	|-----------------------O-----------------------|

**Diabetes mellitus**	|------------------------O-----------------------|
**enge Kleidung (im Bestrahlungsareal)**	|------------------------O-----------------------|
**fortgeschrittenes Krankheitsstadium**	|------------------------O-----------------------|
**Fraktionierungsschema (Normo- vs. Hypofraktionierung)**	|------------------------O-----------------------|
**Gesamtdosis [Gy]**	|------------------------O-----------------------|
**Geschlecht**	|------------------------O-----------------------|
**Hauttyp**	|------------------------O-----------------------|
**langjährige und wiederholte UV-Exposition der Haut**	|------------------------O-----------------------|
**postoperatives Serom**	|------------------------O-----------------------|
**Rauchen**	|------------------------O-----------------------|
**Untergewicht**	|------------------------O-----------------------|
**vorbestehende Hauterkrankungen (bspw. Psoriasis, atopische Dermatitis)**	|------------------------O-----------------------|
**Vorbestrahlung (im gleichen Areal)**	|------------------------O-----------------------|

12 - Wie häufig erfassen Sie in Ihrer Klinik/Praxis den Schweregrad der Radiodermatitis Ihrer Patienten während der Behandlung?

☐ gar nicht
☐ nur im Bedarfsfall
☐ 1x/Woche
☐ 2-5x/Woche
☐ bei Abschluss der Therapie
anders...

---

13 - Wie erfassen Sie in Ihrer Klinik/Praxis den Schweregrad der Radiodermatitis?

Mehrfachauswahl möglich

☐ subjektive ärztliche Einschätzung, also bspw. "leicht" / "mittel" / "schwer"
☐ NCI CTCAE Schweregrade
☐ RTOG/EORTC Schweregrade

☐ Fotodokumentation bei jedem Patienten mit Radiodermatitis

☐ objektive Messverfahren (wie bspw. Reflexionsphotospektrometrie/ Laser-Doppler-Flussmessung)

Andere:

---

14 - Fragen Sie zudem auch dediziert die von den Patienten empfundenen Symptome (PROs: patient-reported outcomes) ab?

Es geht um patient-reported outcomes

☐ Ja

☐ Nein

Andere?

---

15 - Wie häufig fragen Sie die PROs ab?

☐ nur im Bedarfsfall

☐ 1x / Woche

☐ 2-5x / Woche

☐ nur bei Abschluss der Therapie

Anders?

---

16 - Wie erfassen Sie die patient-reported outcomes (PROs)?

☐ selbst gewählte Skala: z.B. mit leichter / mittlerer / schwerer Symptomatik

☐ NCI PRO-CTCAE

☐ STAT (Skin toxicity assessment tool)

☐ Skindex 16

☐ RISRAS (radiation-induced skin reaction assessment score)

☐ BPI (Brief pain inventory)

Andere Tools bzw. Varianten?

---

17 - Wie schätzen Sie die durchschnittliche Schwere der Radiodermatitis Ihrer Patienten ein?

Skala analog CTCAE mit den Stufen 0-3

	**0**	**I°**	**II°**	**III°**
**Brustpatienten**	○	○	○	○
**HNO-Patienten**	○	○	○	○

18 - Welche Informationen erfassen Sie vor Beginn der Radiatio?

Mehrfachauswahl möglich

☐ Hauttyp, z.B. nach Fitzpatrick (Typ 1-6, keltisch bis schwarz)

☐ Prätherapeutisch verwendete topische Medikamente im Bestrahlungsareal werden stets erfasst.

☐ Prätherapeutisch verwendete topische Medikamente im Bestrahlungsareal werden unregelmäßig erfasst (z.B. bei Vorerkrankungen der bestrahlten Hautareale).

☐ Vor der Radiatio verwendete topische Medikamente im Bestrahlungsareal werden nicht erfasst.
☐ Vorbestehende Hauterkrankungen werden stets abgefragt und erfasst.
☐ Vorbestehende Hauterkrankungen werden unregelmäßig erfasst (z.B. bei Vorerkrankungen der bestrahlten Hautareale).
☐ Vorbestehende Hauterkrankungen werden nicht konkret erfasst.

---

19 - Welche Empfehlungen geben Sie Ihren Patienten zur Körperpflege (Waschen/Duschen/Baden) unter Therapie?
Mehrfachauswahl möglich

☐ Waschen/Duschen/Baden soll über den Zeitraum der Radiatio unterlassen werden.
☐ Waschen/Duschen ist gestattet mit gewöhnlichen Seifen, Duschgels oder Shampoos.
☐ Waschen/Duschen ist gestattet mit milden, duftstofffreien und pH-hautneutralen Seifen.
☐ Ausgiebiges bzw. langes Duschen/Baden mit heißem Wasser sollte unterlassen werden.
☐ Ausgiebige Vollbäder mit heißem Wasser sind gestattet.
☐ Schwimmen in gechlortem Wasser oder Salzwasser ist erlaubt.
☐ Sonnenbaden ist auch im Bestrahlungsareal möglich.
☐ Sonnenbaden sollte lediglich im Bereich des Bestrahlungsareals vermieden werden.
☐ Sonnenbaden sollte während der Radiatio vollständig unterlassen werden.
☐ Saunabesuche sind gestattet.
☐ Saunabesuche sollten erst mit Einsetzen einer Radiodermatitis unterlassen werden.
☐ Saunabesuche sollten während der Radiatio vollständig unterlassen werden.
☐ Das Auftragen einer Creme oder Lotion direkt vor Bestrahlung ist erlaubt.
☐ Wärmepackungen (z.B. Fangotherapie i.R. von Wellnessprogrammen) sind erlaubt.
☐ Kühlen ist erlaubt.

---

20 - Was empfehlen Sie im Hinblick auf eine Deodorant- bzw. Antitranspirant-Verwendung?
Mehrfachauswahl möglich

☐ Die Verwendung von Deodorant/Antitranspirant sollte während der Radiatio gänzlich unterlassen werden.
☐ Deodorant/Antitranspirant MIT Alkohol UND Aluminium kann angewendet werden.
☐ Deodorant MIT Alkohol kann angewendet werden.
☐ Antitranspirant MIT Aluminium kann angewendet werden.
☐ Deodorant/Antitranspirant kann auch bei einsetzender/bestehender Radiodermatitis angewendet werden.
☐ Deodorant/Antitranspirant soll bei einsetzender/bestehender Radiodermatitis nicht mehr angewendet werden.

---

21 - Was sollte zur mechanischen Schonung der Haut im Bestrahlungsareal vermieden werden?
Mehrfachauswahl möglich

☐ Reiben auf der Haut (Beispiel: kräftiges Abtrocknen)
☐ eng anliegende Kleidungsstücke über bestrahlter Haut
☐ raue, juckende Kleidungsstücke
☐ Heftpflaster, Schmuck
☐ Massagen an der Stelle der Bestrahlung
☐ Lymphdrainagebehandlungen im Bestrahlungsareal
☐ langes Duschen (länger als 2 Minuten)

22 - Schneiden Sie das PTV (Zielvolumen) von der Haut ab, um die Haut zu schonen?
Natürlich nur, wenn die Haut kein GTV oder CTV darstellt...

◯ ja, 1mm
◯ ja, 2mm
◯ ja, 3mm
◯ ja, 4mm
◯ ja, 5mm
◯ ja, >5mm
◯ nein
Andere:

---

23 - Welche Produkte bzw. Wirkstoffe empfehlen Sie zur topischen Prophylaxe (also vor Auftreten) der Radiodermatitis?
Keine Angst, hier haben wir für Sie nur eine Produktpalette zusammengestellt, aus der Sie bequem auswählen können! Es geht um die topische ***Prophylaxe****. Mehrfachnennungen sind möglich. Sollte etwas nicht aufgeführt sein, ergänzen Sie bitte das fehlende Produkt/Wirkstoff unter "Andere".*

- ☐ keine
- ☐ Aqueous® Cream BP
- ☐ Lanolin, Wollwachs oder -fett
- ☐ Dexpanthenol
- ☐ Urea
- ☐ synthetische Gerbstoffe (z.B. Tannolact®)
- ☐ Puder
- ☐ Cortisonhaltige Topica
- ☐ Calendula-Extrakt (Ringelblume)
- ☐ Biafine® Emulsion Cream
- ☐ Hyaluronsäure
- ☐ Silbersulfadiazin (z.B. Flammazine® Creme)
- ☐ Sucralfat Creme
- ☐ MA 5065D (Xclair® Creme)
- ☐ Gentanviolett-Lösung
- ☐ R1/R2
- ☐ Folienverbände
- ☐ "Sprühpflaster" (z.B. Cavilon von 3M)
- ☐ fetthaltige Topica (z.B. Linola®-N)
- ☐ Aloe Vera
- ☐ Low-level Laserbehandlung

Ihnen fällt noch etwas anderes ein?:

---

24 - Welche topischen Corticosteroide verordnen Sie zur Prophylaxe der Radiodermatitis?

Falls ja, kreuzen Sie bitte das entsprechende Produkt an bzw. ergänzen es unter "Andere".

- ☐ niedrigpotente Glucocorticoide (z.B. Hydrocortison (0,1-1%))
- ☐ mittelpotente Glucocorticoide (z.B. Betagalen® Creme/Salbe)
- ☐ hochpotente Glucocorticoide (z.B. Mometa® Creme/Salbe)

Andere:

---

25 - Verordnen Sie zur Prophylaxe der Radiodermatitis Folienverbände, Verbände oder Sprühpflaster?

Mehrfachauswahl möglich

- ☐ nein
- ☐ Mepitel® film von Mölnlycke
- ☐ Mepilex® lite von Mölnlycke
- ☐ Hydrofilm® von Paul Hartmann
- ☐ Cavilon® No-Sting (Sprühpflaster) von 3M
- ☐ Silber-haltige (Nylon-)Verbände/Auflagen

Andere:

---

26 - Wie effektiv sind Ihrer Meinung nach die folgenden Wirkstoffe für die (topische) Prävention der Radiodermatitis?
Auch hier bitte keine Angst - wir haben hier erneut Produkte aufgelistet, bitte nennen Sie uns Ihre subjektive Einschätzung auf Grundlage Ihrer Erfahrungen. Es geht hier um die ***prophylaktische*** *Behandlung!*

	keine Erfahrung	uneffektiv	eher effektiv	effektiv
keine	○	○	○	○
Aqueous® Cream BP	○	○	○	○
Lanolin, Wollwachs oder -fett	○	○	○	○
Dexpanthenol	○	○	○	○
Urea	○	○	○	○
synthetische Gerbstoffe (z.B. Tannolact®)	○	○	○	○
Puder	○	○	○	○
Calendula-Extrakt (Ringelblume)	○	○	○	○
Biafine® Emulsion Cream	○	○	○	○
Hyaluronsäure	○	○	○	○
Silbersulfadiazin (z.B. Flammazine® Creme)	○	○	○	○
Sucralfat Creme	○	○	○	○
MA 5065D (Xclair® Creme)	○	○	○	○
Gentanviolett-Lösung	○	○	○	○
R1/R2	○	○	○	○
Folienverbände	○	○	○	○
"Sprühpflaster" (z.B. Cavilon von 3M)	○	○	○	○
fetthaltige Topica (z.B. Linola®-N)	○	○	○	○
Aloe Vera	○	○	○	○
niedrigpotentente Glucocorticoide	○	○	○	○
mittelpotente Glucocorticoide	○	○	○	○
hochpotentente Glucocorticoide	○	○	○	○

**Low-level Laserbehandlung**	◯	◯	◯	◯

27 - Welche Produkte bzw. Wirkstoffe empfehlen Sie zur systemischen Prophylaxe der Radiodermatitis?
Bitte geben Sie im Textfeld die Dosierung des von Ihnen empfohlenen Wirkstoffs/Produkts an

☐ keine
☐ Sucralfat Granulat
☐ Acetylsalicylsäure (ASS)
☐ Wobe-Mugos (Enzymmischung)
☐ Zink
☐ Pentoxyfillin
☐ Vitamin C hochdosiert
☐ Vitamin E hochdosiert
Dosierung? Oder doch etwas anderes?

---

28 - Wie effektiv sind Ihrer Meinung nach die folgenden Wirkstoffe für die (systemische) Prävention der Radiodermatitis?
Es geht hier um die systemische Einnahme der unten genannten Wirkstoffe. Bitte nennen Sie uns Ihre subjektive Einschätzung auf Grundlage Ihrer Erfahrung

	**keine Erfahrung**	**uneffektiv**	**eher effektiv**	**effektiv**
**Sucralfat Granulat**	◯	◯	◯	◯
**Acetylsalicylsäure (ASS)**	◯	◯	◯	◯
**Wobe-Mugos (Enzymmischung)**	◯	◯	◯	◯
**Zink**	◯	◯	◯	◯
**Pentoxyfillin**	◯	◯	◯	◯
**Vitamin C hochdosiert**	◯	◯	◯	◯
**Vitamin E hochdosiert**	◯	◯	◯	◯

29 - Was ist Ihre Entscheidungsgrundlage, eine Prophylaxe sowohl topisch als auch systemisch durchzuführen?
Bitte geben Sie uns Ihre subjektive Einschätzung

	**nicht wichtig**	**eher unwichtig**	**eher wichtig**	**wichtig**
**klinikinterne SOP (sofern vorhanden)**	◯	◯	◯	◯

**persönliche Behandlungserfahrungen**	○	○	○	○
**aktuelle Literatur/Publikationen**	○	○	○	○
**Empfehlung von Kollegen anderer Kliniken/Praxen**	○	○	○	○
**Leitlinie Supportive Therapie bei onkologischen Patienten**	○	○	○	○
**Mitteilungen über neue Produkte der Pharmaindustrie/Pharmavertreter und erhaltene "Muster"**	○	○	○	○

30 - Empfehlen Sie Ihren Patienten zur Prophylaxe und Therapie der Radiodermatitis auch alternative Behandlungsverfahren?
Wenn wir etwas nicht aufgeführt haben sollten, zögern Sie nicht, uns Ihre Ideen und Erfahrungen als Freitext einzugeben!

☐ nein
☐ Verwendung von "Hausmitteln" (bspw. gekühlte Umschläge mit Quark, gekühlte Umschläge mit schwarzem Tee etc.)
☐ Homöopathie
☐ Traditionelle Chinesische Medizin
☐ Akupunktur
☐ Ganzkörperkältetherapie (z.B. in Eiskabine bei -160°C für wenige Minuten)
Andere Vorschläge? Nur zu, bitte geben Sie uns Ihre Empfehlung!

---

31 - Nun geht es um die Methoden/Strategien zur Therapie der bereits bestehenden Radiodermatitis (Management-Strategien)!
32 - Welche Wirkstoffe bzw. Produkte empfehlen Sie zur topischen Anwendung bei bestrahlungsassoziiertem Erythem mit und ohne trockene Desquamation?
*Suchen Sie es sich bequem aus der nachstehenden Liste: Es geht um topisch verwendete Substanzen beim bestrahlungsassoziierten Erythem und/oder trockener Desquamation entsprechend **Grad 1 und 2 CTCAE**. Mehrfachnennungen sind möglich.*
☐ keine
☐ Aqueous® Cream BP
☐ Lanolin, Wollwachs oder -fett
☐ Dexpanthenol
☐ Urea
☐ synthetische Gerbstoffe (z.B. Tannolact®)
☐ Puder
☐ Cortisonhaltige Topica
☐ Calendula-Extrakt (Ringelblume)
☐ Biafine® Emulsion Cream
☐ Hyaluronsäure

☐ Silbersulfadiazin (z.B. Flammazine® Creme)
☐ Sucralfat Creme
☐ MA 5065D (Xclair® Creme)
☐ Gentanviolett-Lösung
☐ R1/R2
☐ fetthaltige Topica (z.B. Linola®-N)
☐ Aloe Vera
☐ silberbeschichtete Wundauflage (silver nylon dressing)
☐ feuchte Umschläge mit nicht traumatisierenden Kompressen und antiseptischer Lösung (z.B. 2-3x/Tag für 20 Min)
☐ trockene Wundverbände (Schaumstoffe aus z.B. Polyurethan, Calciumalginatwatten bzw. –kompressen)
☐ Folienverbände bzw. Filme
☐ Low-Level-Lasertherapie
Andere Vorschläge? Nur zu, bitte geben Sie uns Ihre Empfehlung!

---

33 - Für wie effektiv beurteilen Sie folgende topische Produkte bzw. Wirkstoffe zur Behandlung eines Strahlenerythems mit und ohne trockene Desquamation (I° und II° CTCAE).
*Bitte nicht erschrecken! Es geht um topisch verwendete Substanzen beim bestrahlungsassoziierten Erythem und/oder trockene Desquamation entsprechend **Grad 1 und 2 CTCAE**. Mehrfachnennungen möglich*

	keine Erfahrung	uneffektiv	eher effektiv	effektiv
keine	◯	◯	◯	◯
Aqueous® Cream BP	◯	◯	◯	◯
Lanolin, Wollwachs oder -fett	◯	◯	◯	◯
Dexpanthenol	◯	◯	◯	◯
Urea	◯	◯	◯	◯
synthetische Gerbstoffe (z.B. Tannolact®)	◯	◯	◯	◯
Puder	◯	◯	◯	◯
Cortisonhaltige Topica	◯	◯	◯	◯
Calendula-Extrakt (Ringelblume)	◯	◯	◯	◯
Biafine® Emulsion Cream	◯	◯	◯	◯
Hyaluronsäure	◯	◯	◯	◯
Silbersulfadiazin (z.B. Flammazine® Creme)	◯	◯	◯	◯
Sucralfat Creme	◯	◯	◯	◯

**MA 5065D (Xclair® Creme)**	◯	◯	◯	◯
**Gentanviolett-Lösung**	◯	◯	◯	◯
**R1/R2**	◯	◯	◯	◯
**fetthaltige Topica (z.B. Linola®-N)**	◯	◯	◯	◯
**Aloe Vera**	◯	◯	◯	◯
**silberbeschichtete Wundauflage (silver nylon dressing)**	◯	◯	◯	◯
**feuchte Umschläge mit nicht traumatisierenden Kompressen und antiseptischer Lösung (z.B. 2-3x/Tag für 20 Min)**	◯	◯	◯	◯
**trockene Wundverbände (Schaumstoffe aus z.B. Polyurethan, Calciumalginatwatten bzw. –kompressen)**	◯	◯	◯	◯
**Folienverbände bzw. Filme**	◯	◯	◯	◯
**Low-Level-Lasertherapie**	◯	◯	◯	◯

34 - Abschließend kommen wir noch zur Versorgung bei Patienten mit(!) feuchter Desquamation. Bevorzugen Sie die "trockene" oder "feuchte" Versorgung?

◯ trocken
◯ feucht
◯ Bei Patienten mit feuchter Desquamation bei Bestrahlung wird die Wundversorgung durch andere Disziplinen (z.B. Dermatologen, Hausärzte) übernommen.
Anderes Vorgehen:

---

35 - Empfehlen Sie bei Radiodermatitis II° und III° mit(!) feuchter Desquamation ohne Verdacht auf eine kutane Infektion ein Oberflächenantiseptikum?
Es geht um die Radiodermatitis Grad 2 und 3 ***mit*** *feuchter Desquamation. Ist ein Antiseptikum indiziert?*
◯ nein
◯ ja

36 - Was ist Ihr bevorzugtes Oberflächenantiseptikum?

37 - Sie wundversorgen Ihre Patienten "trocken". Was ist Ihr übliches Vorgehen, wenn keine(!) Infektionszeichen vorliegen?
Wie schätzen Sie die nachstehenden Therapiestrategien hinsichtlich Effektivität und Sicherheit ein?

	keine Erfahrung	uneffektiv	eher effektiv	effektiv
Kompressen	◯	◯	◯	◯
Wunddistanzgitter/wirkstofffreie Wundgazen	◯	◯	◯	◯
Wundschnellverbände/"Pflaster"	◯	◯	◯	◯
Puder	◯	◯	◯	◯
Gentianaviolett-Lösung	◯	◯	◯	◯
Cortisonhaltige Topica	◯	◯	◯	◯
Dexpanthenolhaltige Topica	◯	◯	◯	◯
Oberflächenantiseptikum	◯	◯	◯	◯
Lokalantibiotikum	◯	◯	◯	◯

38 - Ihre Wundversorgung bei Desquamation erfolgt "feucht".
Wie schätzen Sie die nachstehenden Therapiestrategien hinsichtlich Effektivität und Sicherheit ein?

	keine Erfahrung	uneffektiv	eher effektiv	effektiv
Kompressen	◯	◯	◯	◯
Wunddistanzgitter/wirkstofffreie Wundgazen	◯	◯	◯	◯
Semipermeable Folienverbände	◯	◯	◯	◯
Polyurethan-Schaumverbände	◯	◯	◯	◯
Hydrogelkompressen	◯	◯	◯	◯
Hydrocolloidverbände	◯	◯	◯	◯
hochreinigende Polyacrylat-Wundauflagen	◯	◯	◯	◯
Cortisonhaltige Topica	◯	◯	◯	◯
Urea-Lotion 2-4%ig	◯	◯	◯	◯
Urea-Lotion 5-10%ig	◯	◯	◯	◯
Synthetische Gerbstoffe (z.B. Tannin-Gel, TannoLact)	◯	◯	◯	◯

**Sprühschäume**	○	○	○	○

39 - Wie viel Hoffnung haben Sie in neue Therapien hinsichtlich einer Verbesserung der Radiodermatitis?
Hier können Sie einen Stern (links, bei wenig Optimismus) bis vier Sterne (rechts, bei großem Optimismus) verteilen!

	|-----------------------O-----------------------|
