# Supplementary material for: Risk assessment, surveillance, and nonpharmaceutical prevention of acute radiation dermatitis: results of a multicentric survey among the German-speaking radiation oncology community
Source: Strahlenther Onkol. 2023 Apr 26;199(10):891–900. doi: 10.1007/s00066-023-02074-w (PMC10542714; doi:10.1007/s00066-023-02074-w)
Supplement: Supplementary file 2 — Supplementary Table 1: Members of the GRDSG [file 66_2023_2074_MOESM2_ESM.docx]

Suppl. Table 1: Members of the GRDSG

German Radiation Dermatitis Survey Group
(GRDSG)

| Ali | Munef | Department of Radiation Therapy, Johanniter Hospital | Stendal | DE |
| --- | --- | --- | --- | --- |
| Alshammas | Nizar | Department of Radiation Oncology, Clinic of Chemnitz | Chemnitz | DE |
| Appel | Wiebke | Outpatient clinic of Radiation Therapy | Neuwied | DE |
| Arnold | Christian | Department of Radiation Therapy and Oncology, University Hospital Frankfurt | Frankfurt | DE |
| Astner | Sabrina | Radiation Therapy Munich Nymphenburg and Fürstenfeldbruck | Munich | DE |
| Attassi | Mared | Radiation Therapy 360° Marien-Hospital of Aachen | Aachen | DE |
| Baumann | René | Department of Radiation Oncology, St. Marien-Hospital | Siegen | DE |
| Becker-Schiebe | Martina | Radiation Therapy & Radiation Oncology, Municipal Hospital | Braunschweig | DE |
| Berkovic | Katharina | Clinic of Radiation Therapy | Essen | DE |
| Bischoff | Annett | Outpatient clinic of Radiation Therapy | Leipzig | DE |
| Bohrer | Markus | Clinic of Radiation Therapy Mannheim | Mannheim | DE |
| Born | Claudia | Department of Radiation Therapy and Palliative Medicine, Marien-Hospital Stuttgart | Stuttgart | DE |
| Buschbeck | Britta | Outpatient clinic of Radiation Therapy Prüner Gang | Kiel | DE |
| Chizzali | Barbara | Outpatient clinic of Radiation Therapy of Rosenheim | Ebersberg | DE |
| Crasselt | Frederik | RNS Clinic of Radiation Oncology | Wiesbaden | DE |
| Dietzel | Christian | Department of Radiation Therapy, University Clinic Halle (Saale) | Halle (Saale) | DE |
| Dima | Gabriela | Clinic of Radiation Therapy | Mühlhausen | DE |
| Ebersberger | Anne | Center of Radiation Oncology Dr. Staab | Bad Kreuznach | DE |
| Eczko | Julia-Christin | Department of Radiation Therapy and Radiation Oncology St. Georg-Hospital | Leipzig | DE |
| Exeli | Lukas | Radiation Therapy Frankfurt | Frankfurt | DE |
| Falter | Bernhard | Outpatient clinic of Radiation Therapy Cologne | Cologne | DE |
| Feißt | Andreas | Outpatient clinic of Radiology Neuwied | Neuwied | DE |
| Gross | Markus | Department of Radiation Therapy and Radiation Oncology University Hospital Basel | Basel | CH |
| Güßbacher | Christoph | Xcare Radiation Therapy Diakonie Hospital | Neunkirchen | DE |
| Härtel | Christina | Department of Radiation Oncology, Cyberknife- und Radiation Therapy University Hospital Cologne | Cologne | DE |
| Hartmann | Claudia | Outpatient clinic of Radiation Therapy Taunus GmbH | Bad Homburg | DE |
| Heiming | Marcus | Department of Radiation Oncology, Cyberknife- und Radiation Therapy, University Hospital Cologne | Cologne | DE |
| Heinrich | Christine | Outpatient clinic of Radiation Therapy, Agatharied GmbH | Hausham | DE |
| Hering | Kathrin | Department of Radiation Oncology, University Hospital Leipzig | Leipzig | DE |
| Hermann | Robert Michael | Clinic of Radiation Therapy and Radiation Oncology | Westerstede | DE |
| Hoehle | Frieder | Radiation Therapy Bonn-Rhein-Sieg | Bonn | DE |
| Hoffmann | Christian | Department of Radiation Therapy, University Hospital Essen | Essen | DE |
| Holy | Richard | Radiation Therapy 360° Marien-Hospital of Aachen | Aachen | DE |
| Jacob | Ingrid | Radiation Therapy and Radiation Oncology Hospital of Traunstein | Traunstein | DE |
| Jahn | Franziska | Karl-Lennert-Cancer-Center, University Hospital Schleswig-Holstein | Kiel | DE |
| Janssen | Stefan | Department of Radiation Therapy, University Hospital Schleswig-Holstein | Luebeck | DE |
| Jazmati | Danny | Westdeutsches Protonentherapiezentrum, University Hospital Essen | Essen | DE |
| Joos | Susanne | Department of Radiation Oncology und Radiaton Therapy, Alb Fils Hospital GmbH | Göppingen | DE |
| Kahl | Klaus-Henning | Department of Radiation Therapy and Radiation Oncology, University Hospital Augsburg | Augsburg | DE |
| Kipping | Andrea | Department of Radiation Therapy and Radiation Oncology, SRH Wald-Hospital | Gera | DE |
| Konrad | Stefan | University Hospital of Radiation Oncology, University Hospital Vienna/AKH Vienna | Vienna | AT |
| Koswig | Stephan | Department of Radiation Oncology and Radiation Therapy, Helios Hospital | Bad Saarow | DE |
| Kraus-Tiefenbacher | Uta | Outpatient clinic of Radiation Therapy, Nordwest Hospital gmbH | Frankfurt | DE |
| Krause | Stefan | Radiation Therapy, Hospital of Fulda gAG | Fulda | DE |
| Krebs | Heike | Department of Radiation Oncology, Helios Hospital Berlin-Buch | Berlin | DE |
| Kunellis | Eva | Department of Radiation Therapy, Evangelical Hospital Hamm | Hamm | DE |
| Linde | Philipp | Department of Radiation Oncology, Cyberknife- und Radiation Therapy, University Hospital Cologne | Cologne | DE |
| Lipponer | Susanne | Institut of Radiation Therapy Dr. von Essen | Koblenz | DE |
| Loibnegger | Karin | Radiation Oncology and Radiation Therapy, State Hospital Feldkirch | Feldkirch | AT |
| Löschcke | Michael | Outpatient clinic of Radiation Therapy and Nuclear Medicine | Cologne-Merheim | DE |
| Lütter | Christiana | Outpatient clinic of Radiation Therapy GmbH | Cologne | DE |
| Melchior | Patrick | Department of Radiation Therapy and Radiation Oncology, University Hospital Saarland | Bad Homburg | DE |
| Melzer | Wolfgang | Outpatient clinic of Radiation Therapy, Aue | Aue | DE |
| Merten | Roland | Department of Radiation Therapy and Oncology, Medizinische Hochschule Hannover | Hannover | DE |
| Momm | Felix | Radiation-Oncology, Ortenau Hospital | Offenburg-Kehl | DE |
| Morell | Nicole | Department of Radiation Therapy and Tumor Medicine, Medius Hospital Ostfildern-Ruit | Ostfildern | DE |
| Mucha | Maja | Department of Radiation Therapy, University Hospital Essen | Essen | DE |
| Müller | Christoph | Department of Radiation Therapy, Radiation Oncology ans Palliative Medicine, ViDia Hospital | Karlsruhe | DE |
| Mutiara | Sally | Department of Radiation Oncology and Radiation Therapy, Charité Berlin | Berlin | DE |
| Nestle | Ursula | Department of Radiation Oncology, Maria Hilf Hospital | Moenchengladbach | DE |
| Nießen | Michael | Radiation Oncology 360°, Praxis in der Ottostraße | Leverkusen | DE |
| Niewald | Marcus | Department of Radiation Therapy and Radiation Oncology, University Hospital Saarland | Bad Homburg | DE |
| Oraei-Abbassian | Faraneh | Department of Radiation Oncology, University Hospital Rostock | Rostock | DE |
| Ott | Jessica | Radiation Oncology, University Hospital Erlangen | Erlangen | DE |
| Panzer | Monika | Department of Radiation Therapy and Radiation Oncology, Weilheim Hospital | Oberbayern | DE |
| Raith | Bettina | RADIO-LOG, Radiation Therapy | Passau | DE |
| Rapp | Matthias | Department of Radiation Therapy and Radiation Oncology, University Hospital Mainz | Mainz | DE |
| Reinartz | Gabriele | Department of Radiation Therapy and Radiation Oncology, University Hospital Muenster | Muenster | DE |
| Riesenbeck | Dorothea | Radiation Therapy Reimann/Riesenbeck | Recklinghausen | DE |
| Riggenbach | Elena | University Hospital of Radiation Oncology, Inselspital Bern | Bern | CH |
| Roeder | Falk | University Hospital of Radiation Therapy and Radiation Oncology, Paracelsus Medical Private University | Salzburg | AT |
| Rudykina | Anna | Center of Radiation Therapy Rheine-Osnabrück | Rheine | DE |
| Schleicher | Ursula M. | Clinic of Radiation Therapy, Dueren | Dueren | DE |
| Schmidt | Franziska | Department of Radiation Oncology, Helios Hospital Erfurt | Erfurt | DE |
| Schmitter | Charlotte | Radiation Oncology, University Hospital Erlangen | Erlangen | DE |
| Schmitz | Susanne | Department of Raditaion Oncology, Maria Hilf Hospital | Moenchengladbach | DE |
| Schultze | Jürgen | Karl-Lennert-Cancer-Center, University Hospital Schleswig-Holstein | Kiel | DE |
| Schulze | Katrin | Radiation Therapy, Hospital of Fulda gAG | Fulda | DE |
| Semrau | Sabine | Radiation Oncology, University Hospital Erlangen | Erlangen | DE |
| Sester | Susanne | University Hospital of Radiation Oncology, Inselspital Bern | Bern | CH |
| Soborun | Vicky | Department of Radiation Therapy and Radiation Oncology, University Hospital Carl Gustav Carus | Dresden | DE |
| Sperk | Elena | Mannheim Cancer Center, University Hospital Heidelberg | Mannheim | DE |
| Stanek | Christian | Institut of Radiation Oncology, Hospital Ottakring | Vienna | AT |
| Timke | Carmen | Clinic of Radiation Therapy, Malteser Hospital St. Franziskus Hospital | Flensburg | DE |
| Trommer | Maike | Department of Radiation Oncology, Cyberknife- und Radiation Therapy, University Hospital Cologne | Cologne | DE |
| Tschirner | Sabine | Radiation Therapy Bonn-Rhein-Sieg | Bonn | DE |
| Wegen | Simone | Department of Radiation Oncology, Cyberknife- und Radiation Therapy, University Hospital Cologne | Cologne | DE |
| Welzel | Simone | Department of Radiation Therapy and Radiation Oncology, Pius-Hospital | Oldenburg | DE |
| Winkhaus | Frauke | Department of Radiation Therapy and Palliative Medicine, Marien-Hospital Stuttgart | Stuttgart | DE |
| Wollseifer | Michael | Outpatient clinic of Radiation Therapy Cologne | Cologne | DE |
| Zimmermann | Jörg | Outpatient clinic of Radiation Therapy Alstertal | Hamburg | DE |
| Zurabashvili | Mariam | Department of Radiation Therapy and Radiation Oncology, Hospital Schwarzwald-Baar | Villingen-Schwenningen | DE |

We thank all the members of the GRDSG as well as all of the other participants for their support.
