# Supplementary figures and images for: Risk assessment, surveillance, and nonpharmaceutical prevention of acute radiation dermatitis: results of a multicentric survey among the German-speaking radiation oncology community
Source: Strahlenther Onkol. 2023 Apr 26;199(10):891–900. doi: 10.1007/s00066-023-02074-w (PMC10542714; doi:10.1007/s00066-023-02074-w)

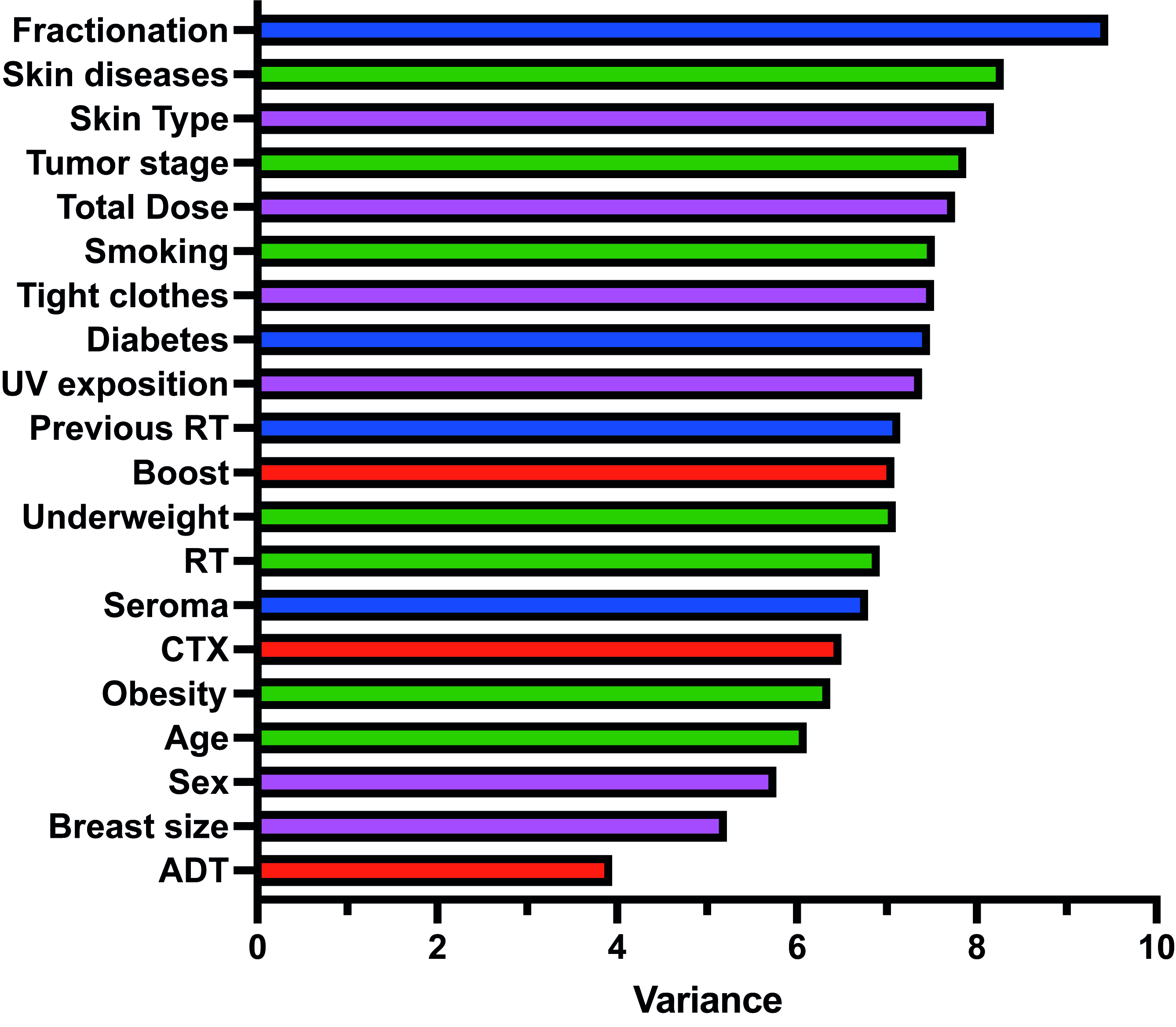

Supplement: Supplementary file 3 — Supplementary Fig. 1: Variance in impact of risk factors on radiation dermatitis color-coded by treatment-associated (red), invariable (green), lifestyle (purple) and RT-dependent (blue) risk factors. Abbreviations: ADT = androgen deprivation therapy; CTX = chemotherapy; RD = radiation dermatitis; RT = radiotherapy. [file 66_2023_2074_MOESM3_ESM.tif]

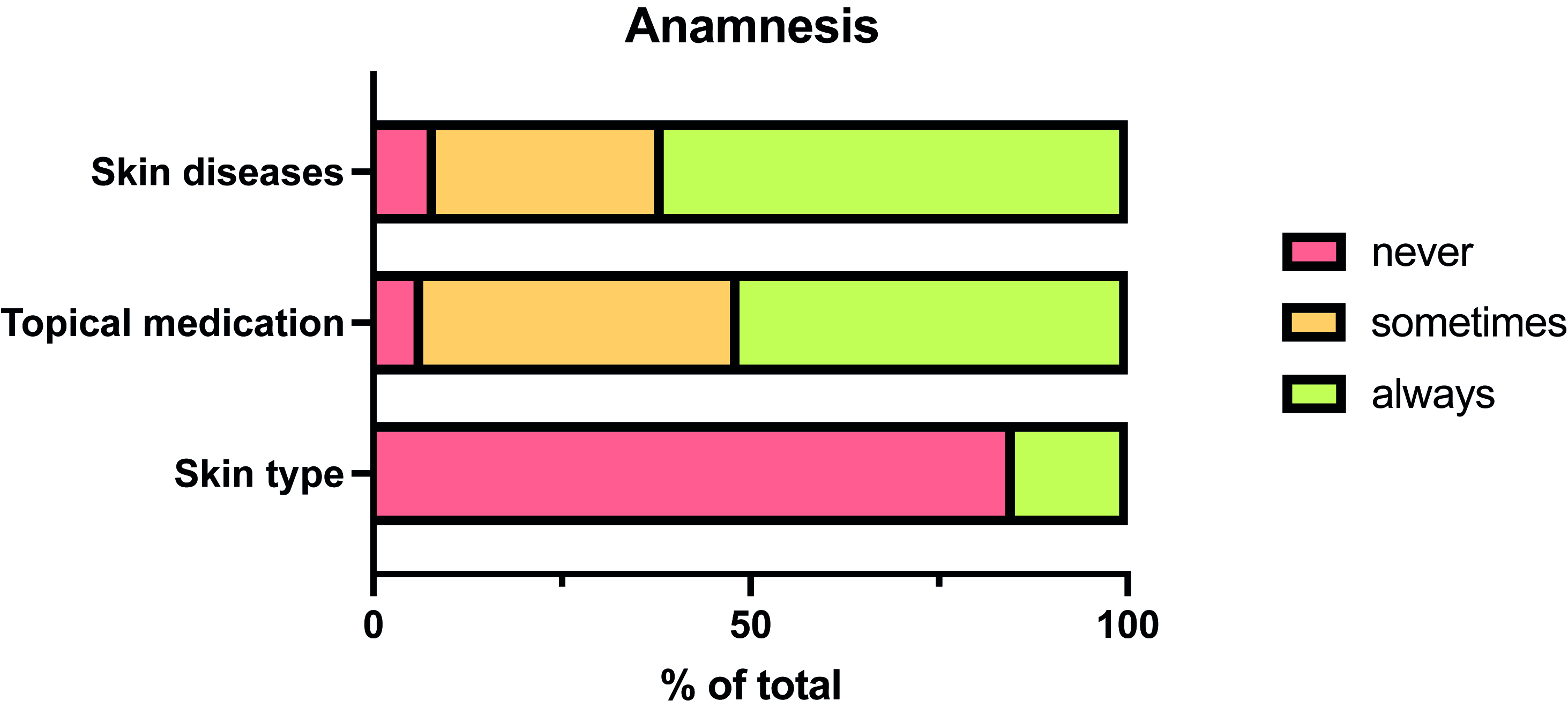

Supplement: Supplementary file 4 — Supplementary Fig. 2: Radiation dermatitis-relevant anamnesis. [file 66_2023_2074_MOESM4_ESM.tif]
